# Isolable Phosphanylidene Phosphorane with a Sterically Accessible Two-Coordinate Phosphorus Atom**

**DOI:** 10.1002/anie.201204998

**Published:** 2012-09-05

**Authors:** Brian A Surgenor, Michael Bühl, Alexandra M Z Slawin, J Derek Woollins, Petr Kilian

**Affiliations:** EaStChem School of Chemistry, University of St AndrewsSt Andrews Fife KY16 9ST (UK); * EaStChem School of Chemistry, University of St AndrewsSt Andrews Fife KY16 9ST (UK)

**Keywords:** density functional calculations, donor-acceptor systems, multiple bonds, phosphorus, structure elucidation

Phosphanylidene-σ^4^-phosphoranes (RP=PR′_3_) are phosphorus analogues of alkylidene-σ^4^-phosphoranes (R_2_C=PR′_3_), better known as Wittig reagents. Phosphanylidene-σ^4^-phosphoranes are synthetically accessible in the free form (RP=PR′_3_) and also in the transition-metal-stabilized form (L_n_M←P(R)=PR′_3_).^1^ The latter complexes are commonly used in both P=C bond generation and as a source of the phosphinidene (R-P) moiety in the continuing pursuit of new terminal phosphinidene complexes R-P=ML_*n*_. In marked contrast, free phosphanylidene-σ^4^-phosphoranes have received little attention because isolable (i.e., thermally stable) examples remain rare.^2^–^4^ Herein we report the synthesis and structure of the stable cyclic phosphanylidene-σ^4^-phosphorane **3** (see Scheme 1). Compound **3** possesses a sterically nonhindered phosphanylidene moiety, yet it is thermally stable enough to be isolated and stored at room temperature. The potential diversity of the coordination chemistry of **3** is illustrated by its transition-metal (Pd^0^) complex and bis(borane) adduct.

Recently, we synthesized the first “bottleable” (i.e., room temperature stable) phosphine–phosphine donor–acceptor (DA) complex **1**.^5^ Because of their normally low thermal stability, the reactivity of phosphine–phosphine complexes (other than thermal decomposition pathways) is virtually unknown. Having access to **1**, we set off to investigate its chemistry in detail. We were especially intrigued by the possibility of utilizing **1** as a precursor for compounds with a low-coordinate phosphorus atom in the *peri* position, since no compounds of this type had been reported in the literature.^6^ We postulated that the specific *peri* arrangement of the relatively basic P*i*Pr_2_ group and the reactive dihalophosphine group should make **1** a good source of “across the *peri* gap” donor-stabilized, low-valent phosphorus species.

The initial reactivity screen of **1** towards reducing reagents revealed that the reaction with BH_3_⋅Me_2_S proceeded cleanly, giving the bis(borane) adduct **2** (*δ*_P_=13.6 and 43.9 ppm, ^1^*J*_PP_=198.5 Hz) in almost quantitative yield (Scheme 1). Although **2** does not possess a low-coordinate phosphorus center, the single-crystal X-ray structure (Figure 1)^7^ confirmed an interesting bonding situation in which a Lewis-base-stabilized phosphinidene moiety acts as a double donor towards two Lewis-acidic (borane) moieties. Two representative resonance forms of **2** (zwitterionic and with DA bonding) are shown in Scheme 2. The P1–P2 distance in **2** (2.2208(11) Å), is consistent with a P–P single bond. The P2–B1 and P2–B2 distances [1.943(5) and 1.943(4) Å, respectively] are equal and are also as expected for P–B single bonds (typical range 1.90 to 1.95 Å). As mentioned recently by Protasiewicz and co-workers,2d whilst of fundamental interest, no bis(borane) push-pull phosphinidene systems have been structurally characterized and compound **2** thus represents the first example. Notably, the attempt by Protasiewicz and co-workers to generate the analogous bis(borane) adduct from the sterically encumbered phosphanylidene phosphorane (2,6-Mes_2_C_6_H_3_)-P=PMe_3_ **5 a** failed and only the monoborane adduct **5 a**⋅BH_3_ was isolated and spectroscopically characterized.2b,d Also, Bertrand and co-workers report only the monoborane adduct **6**⋅BH_3_ upon reaction of the parent crowded cyclic phosphanylidene-σ^4^-phosphorane **6** with BH_3_⋅SMe_2_.3d The (spectroscopically characterized) bis(borane) adduct Me_3_P=PCF_3_⋅2 BH_3_ thus represents the only other example of a phosphine-donor-stabilized neutral phosphorus atom acting as a double donor to two borane units.^8^ In contrast, several examples of carbene-stabilized phosphorus bis(borane) adducts were reported recently in the literature.^9^


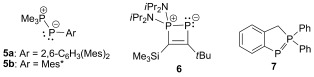


**Figure 1:**
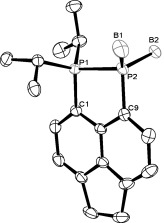
Molecular structure of **2**. Thermal ellipsoids are set at 40 % probability; hydrogen atoms are omitted for clarity.

**Scheme 1:**
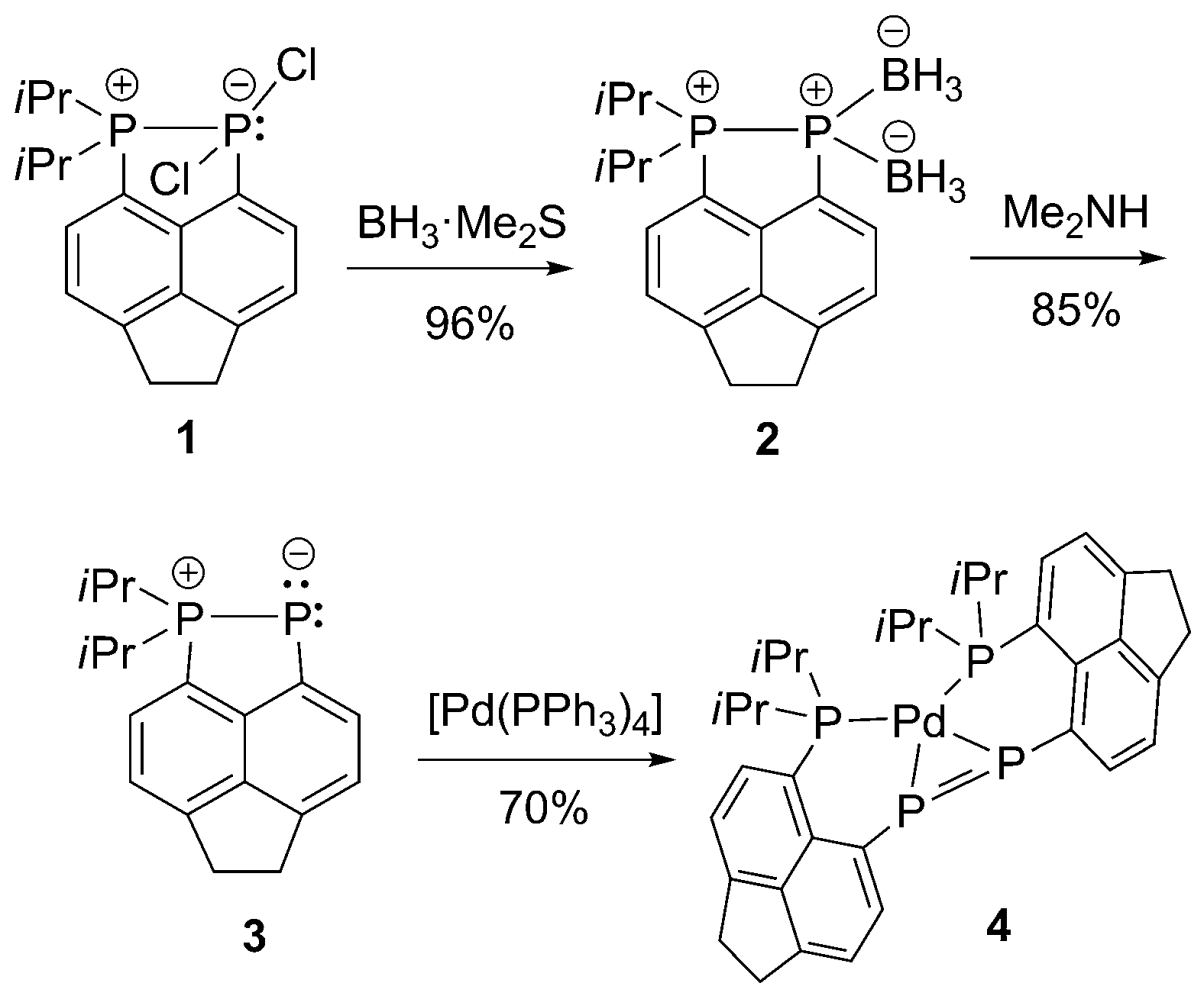
Synthesis and selected reactivity of the phosphanylidene phosphorane **3**.

**Scheme 2:**
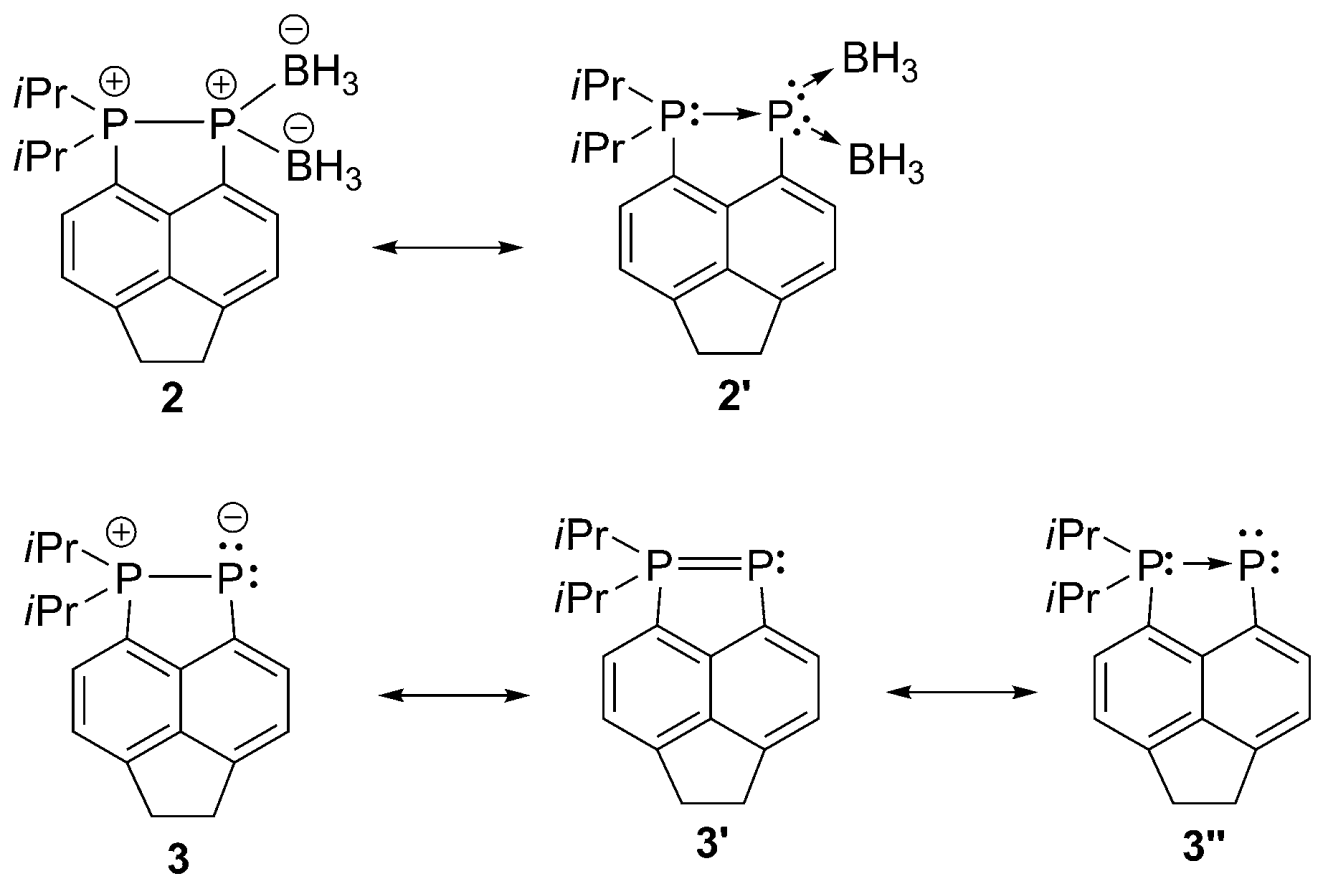
Resonance structures of **2** and **3**.

To assess the bonding in the bis(borane) species we calculated the borane dimethyl sulfide (BH_3_⋅SMe_2_) addition energies (Δ*E*_add_) to the phosphanylidene phosphoranes **3**, **5 a**, and **6** at the B3LYP-D3 level of density functional theory (Table 1).^10^ The notable driving force of Δ*E*_add_=−14.2 kcal mol^−1^ is computed for the reaction of **3** + 2 BH_3_⋅SMe_2_, whereas significantly smaller Δ*E*_add_ values are obtained for both **5 a** + 2 BH_3_⋅SMe_2_ and **6** + 2 BH_3_⋅SMe_2_ (−6.7 and −6.8 kcal mol^−1^, respectively). The calculated natural charges (*q*) on the phosphanylidene P atoms (B3LYP/6-31+G*) in the series **3**, **5 a**, and **6** show very little variation from −0.1 (Table 1). It is therefore likely that reduced steric shielding of the phosphanylidene unit (rather than electronic factors) is predominantly responsible for the observed increased affinity of **3** towards borane, and Lewis acids in general. The stability of **2** is indeed remarkable, as it shows no signs of decomposition when stored in air as a solid for weeks at room temperature and only very slow decomposition takes place in wet/oxygenated organic solvents.

**Table 1:** Calculated driving force for the addition of 2 BH_3_·SMe_2_, natural charges (*q*), Wiberg bond indices (WBIs), and optimized parameters for compounds 3, 5 a, and 6. Values in parentheses are those for adducts with two BH_3_ molecules.

Compound Property	3(3⋅2 BH_3_=2)	5 a(5 a⋅2 BH_3_)	6(6⋅2 BH_3_)
Δ*E*_add_ [kcal mol^−1^]^[a]^	−14.2	−6.7	−6.8
*q*(P_phosphanylidene_)^[b]^	−0.08	−0.09	−0.09
*q*(P_phosphorane_)^[b]^	1.25	1.12	1.72
WBI (P–P)^[b]^	1.13 (0.94)	1.16 (0.85)	0.92 (0.77)
P-P-C [°]^[c,d]^	89.5	111.5	70.9
P–P [Å]^[c]^	2.165 (2.260)	2.141 (2.267)	2.184 (2.289)

[a] Reaction energies (Δ*E*_add_) for **X** + 2 BH_3_⋅SMe_2_ → **X**⋅2 BH_3_ + 2 SMe_2_ (**X**=**3**, **5 a**, **6**), including zero-point and BSSE corrections (B3LYP-D3/6-31+G^*^ level). [b] From NBO analysis, B3LYP/6-31+G^*^ level. [c] B3LYP/6-31+G^*^ level. [d] P_phosphorane_-P_phosphanylidene_-C angle.

To obtain the truly low-coordinate species **3**, a borane removal protocol using dimethylamine was applied to **2** (Scheme 1). The phosphanylidene phosphorane **3** was isolated as an intensely red solid in good yield (>80 %). Compound **3** is highly air sensitive, however under inert atmosphere it can be stored without significant signs of decomposition for several weeks even at room temperature. Single-crystal X-ray diffraction confirmed the structure of **3** (Figure 2) with two molecules in the unit cell. The two molecules are almost identical and therefore only one was selected for discussion of the metric parameters. The experimental P1–P2 bond length of 2.148(5) Å is consistent with partial multiple P–P bond character and is significantly shorter than that in **2** (2.2208(11) Å). Indeed the calculated Wiberg bond index (WBI)^11^ between the two P atoms in **3** is slightly larger than 1 (Table 1). The P1-P2-C9 angle (90.4(5)°) is rather acute, and significantly more so than the relevant angle in the only other structurally characterized phosphanylidene phosphorane, **5 a** (106.79(13)°).2b The ^31^P{^1^H} NMR spectrum of **3** consists of an AX system with *δ*_P_=76.7 (*Pi*Pr_2_) and −157.7 ppm (phosphanylidene), with a large magnitude coupling of ^1^*J*_PP_=480 Hz. Notably, the chemical shift of the phosphanylidene group in **3** is remarkably dissimilar to that observed in the four-membered-ring species **6** (*δ*_P_=58.4 ppm),3a but is only slightly shifted to a lower frequency in comparison to those in **5 a** (*δ*_P_=−114.7 ppm) and **5 b** (*δ*_P_=−134.0 ppm).2a A notable shift to a lower frequency is observed in the ^31^P NMR spectra upon deprotection of the phosphanylidene P atom (*δ*_P_=13.6 ppm in **2** versus −157.7 ppm in **3**), and is accompanied by a large increase in the ^1^*J*_PP_ magnitude (from 198.5 in **2** to 480 Hz in **3**).

**Figure 2:**
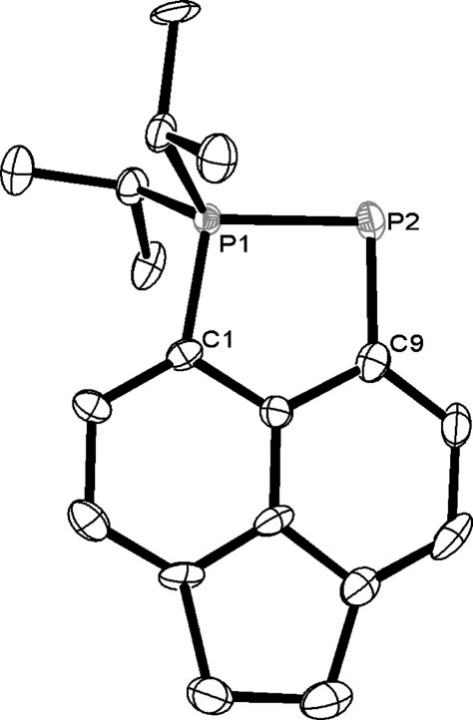
Molecular structure of **3**. Thermal ellipsoids are set at 40 % probability; hydrogen atoms are omitted for clarity.

The ^31^P NMR chemical shift of the phosphanylidene atom in **3** appears at a low frequency for a two-coordinate phosphorus. It is more consistent with the ylide resonance structure **3** which features a high electron density phosphorus center having two lone pairs (Scheme 2). The larger contribution of the ylide resonance form **3** over the doubly bonded form **3′** is further supported by the observation of an elongated P=P bond and the WBI value of 1.13 as discussed above, as well as by additional computational methods (see the Supporting Information).

In 1990 Schmidpeter remarked on the importance of the nature of the substituent attached to the phosphanylidene atom for stability of the neutral phosphanylidene phosphoranes, thus stating that in all known species this substituent is either electron withdrawing or bulky.1b Clearly, incorporation of the phosphanylidene phosphorane moiety into a suitable cyclic system also renders these systems stable. A four-membered-ring skeleton with bulky substituents on the ethylenic moiety is present in the 1,2-diphosphete **6** (isolated by Bertrand and co-workers), and was shown to have a very persistent P–P bond.^3^ In a similar vein, our phosphanylidene phosphorane **3** consists of a C_3_P_2_ ring fused to the rigid acenaphthene moiety, with the *peri*-geometry supporting the P–P bond. However, it is worth noting that to achieve good thermal stability of cyclic phosphanylidene phosphoranes a correct choice of the ring system and of the substituents is necessary. This is illustrated by an early attempt to make a stable phosphanylidene-σ^4^-phosphorane by the formation of an intramolecular P–P bond in **7**. The strategy, utilizing the formation of a five-membered C_3_P_2_ ring, was only partially successful and the desired species **7** was observed by ^31^P NMR spectroscopy, but decomposed below room temperature.^12^

The reactivity of the free phosphanylidene-σ^4^-phosphorane **3** towards [Pd(PPh_3_)_4_] was examined (Scheme 1). The formation of complex **4** accentuates the phosphine–phosphinidene donor–acceptor aspect of bonding in **3** (see resonance structure **3′′** in Scheme 2). In this reaction, the palladium(0) center sequestrates the electron density from the phosphine donor (P1) in a molecule of **3**. The resulting “deprotected” phosphinidene moiety (P2) readily undergoes dimerization to a diphosphene. All four phosphorus atoms of the newly formed (chelating) ligand are coordinated to Pd^0^, two as tertiary phosphine donors, and the remaining two as a side-bonded (η^2^) diphosphene. The crystal structure of **4** is shown in Figure 3. The P1⋅⋅⋅P2 and P3⋅⋅⋅P4 distances (3.244 and 3.188 Å) indicate there is no bonding interaction across the *peri* gap in either of the acenaphthene units, whilst the P2–P3 bond length (2.123(4) Å) is consistent with a P=P double bond elongated upon side-coordination to the metal.^13^, ^14^

**Figure 3:**
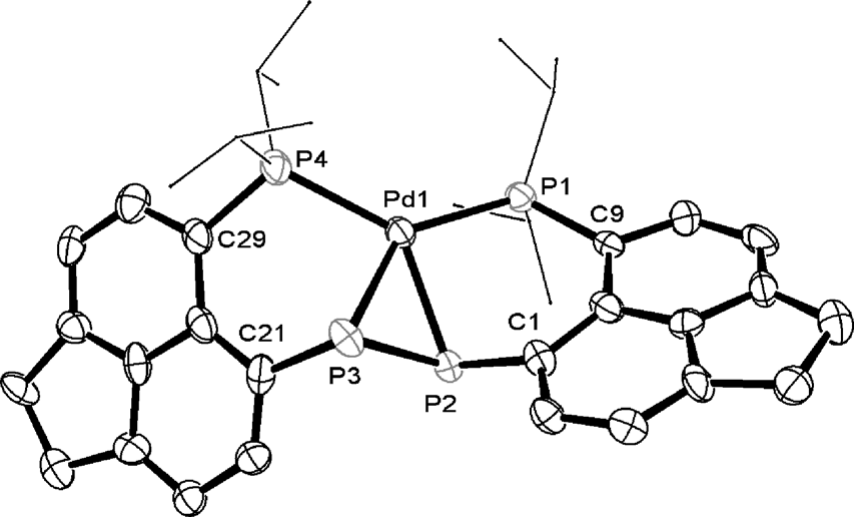
Molecular structure of **4**. Thermal ellipsoids are set at 40 % probability; hydrogen atoms are omitted for clarity. The *i*Pr groups on P1 and P4 (two on each) are drawn as wireframe for clarity.

We have shown that the rigid *peri*-acenaphthene molecular scaffold renders stability to the relatively “naked” two-coordinate phosphanylidene center in **3**, and represents the first example of a low-coordinate phosphorus center directly at the *peri* position of acenaphthene. The compound is also the first example of a phosphorus species with (at least partial) multiple bonding between the two *peri*-atoms. Observation of phosphinidene-like reactivity on coordination of **3** to Pd^0^ provides additional support for the notation that phosphanylidene phosphoranes can indeed be considered main-group complexes of (i.e., base-stabilized) phosphinidenes.1a Our efforts are now directed towards isolation of heavier Group 15 congeners of the species reported herein.

## Supplementary material

Detailed facts of importance to specialist readers are published as ”Supporting Information”. Such documents are peer-reviewed, but not copy-edited or typeset. They are made available as submitted by the authors.
