# Supplementary material for: Isolable Phosphanylidene Phosphorane with a Sterically Accessible Two-Coordinate Phosphorus Atom
Source: Angew Chem Int Ed Engl. 2012 Sep 5;51(40):10150–3. doi: 10.1002/anie.201204998 (PMC3625738; doi:10.1002/anie.201204998)
Supplement: Supplementary file 1 [file anie0051-10150-SD1.pdf]

Angewandte Chemie
Eine Zeitschrift der Gesellschaft Deutscher Chemiker

Supporting Information

© Wiley-VCH 2012

69451 Weinheim, Germany

Isolable Phosphanylidene Phosphorane with a Sterically Accessible Two-Coordinate Phosphorus Atom**

*Brian A. Surgenor, Michael Bühl, Alexandra M. Z. Slawin, J. Derek Woollins, and Petr Kilian**

anie_201204998_sm_miscellaneous_information.pdf

Contents

1 General Procedures and Starting Materials ... 1

2 Experimental Procedures ... 1

2.1 Synthesis of 2 ... 1

2.2 Synthesis of 3 ... 2

2.3 Synthesis of 4 ... 2

3 Crystal Structure Analysis Details ... 3

4 Computational Part ... 4

1 General Procedures and Starting Materials

All experiments were carried out in standard Schlenk glassware or in a glove box with strict exclusion of air and moisture, under a nitrogen or argon atmosphere. Solvents were dried on an MBraun solvent purification system and stored over molecular sieves prior to use. Compound **1** was prepared according to the published procedure.[1] All new compounds were fully characterized by $^{31}P\{^{1}H\}$, ^{1}H and $^{13}C\{^{1}H\}$ NMR, including measurement of $^{1}H\{^{31}P\}$, ^{31}P (^{1}H coupled), H-H DQF COSY, H-P HSQC, H-C HSQC and H-C HMBC experiments. Measurements were performed at 25 °C; 85% H_3PO_4 was used as external standard in ^{31}P, $BF_3{\cdot}OEt_2$ was used as external standard in ^{11}B, TMS as internal in ^{1}H and ^{13}C NMR. Raman spectra were collected on a PE System2000 spectrometer equipped with a near-IR excitation laser. In vacuo refers to a pressure of ca. 10 Pa.

2 Experimental Procedures

2.1 Synthesis of 2

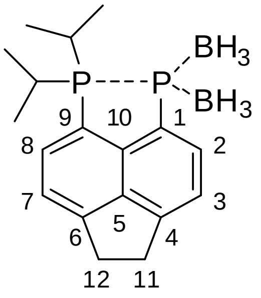

NMR numbering scheme for **2**

To a stirred suspension of **1** (2.0 g, 5.39 mmol) in THF (30 mL), $BH_3{\cdot}Me_2S$ (3.1 mL, 32.7 mmol) was added all at once via syringe, resulting in a yellow solution. After 20 min, the solution was concentrated in vacuo and diethyl ether (20 mL) was added causing a white solid to precipitate. The solid was collected by filtration, washed with diethyl ether (2 × 10 mL) in air and dried in vacuo to give **2** as a colorless powder (1.7 g, 5.18 mmol, 96 %). M.p.: decomp. above 130 °C; Anal. calcd. for $C_{18}H_{28}P_2B_2$ (327.98 g mol^{-1}): C, 65.92; H, 8.60, found: C, 65.95; H, 8.50; ^{1}H NMR (400.1 MHz, $CDCl_3$): δ=0.5–2.0 (br m, 6H; 2×BH_3), 1.36 (dd, ^{3}J(H,P)=18.2 Hz, ^{3}J(H,H)=7.3 Hz, 6H; 2×CH_3), 1.51 (dd, ^{3}J(H,P)=18 Hz, ^{3}J(H,H)=7.2 Hz, 6H; 2×CH_3), 3.1–3.25 (m, 2H; 2×CH at *i*Pr), 3.45–3.54 (m, 2H; 2×CH_2), 7.47–7.5 (m, 1H; H3), 7.51–7.55 (m, 1H; H7), 7.75–7.78 (m, 1H; H2), 7.79–7.82 (m, 1H; H8); $^{13}C\{^{1}H\}$ NMR (67.9 MHz, $CDCl_3$): δ 17.4–17.5 (m(≈t), ^{2}J(C,P)=3.2 Hz, 4×CH_3), 24.5 (d, ^{1}J(C,P)=31.9 Hz, CH at *i*Pr), 30.8 (s, C11), 31.6 (s, C12), 113.1 (d, ^{1}J (C,P)=57.9 Hz, C1), 121.0 (d, ^{3}J(C,P)=10.4 Hz, C7), 122.6 (d, ^{3}J(C,P)=10.1 Hz, C3), 131.7 (dd, ^{2}J(C,P)=12.5 Hz, ^{3}J(C,P)=6.8 Hz, C2), 132.7 (dd, ^{1}J(C,P)=25.2 Hz, ^{2}J(C,P)=9.9 Hz, C9), 133.2 (d, ^{2}J(C,P)=1.6 Hz, C8), 138.2 (br d, ^{2}J(C,P)=18.9 Hz, C10), 138.3–138.5 (m, C5), 147.05 (d, ^{4}J(C,P)=1.3 Hz, C4), 153.9 (d, ^{4}J(C,P)=2.9 Hz, C6); $^{31}P\{^{1}H\}$ NMR (162.0 MHz, $CDCl_3$): δ 13.6 (d, $P(BH_3)_2$), 43.9 (P(*i*Pr)$_2$), ^{1}J(P,P)=201.2 Hz; ^{11}B NMR (128.4 MHz): δ -35.7 to -31.4 (m, 2 × BH_3); IR (KBr disc, cm $^{-1}$): $\tilde{\nu}$=2965 (w), 2907 (m, νC-H), 2368 (s, νB-H), 1612 (m), 1451 (m), 1060 (s), 1036 (vs), 846 (m); Raman (sealed capillary): $\tilde{\nu}$=3062 (m), 2959 (s), 2933 (s), 2908 (s, νC-H), 2370 (m), 2336 (s, νB-H), 1613 (s), 1440 (m), 1385

(m); MS (ES$^+$): *m/z* (%): 351.17 (50) [M+Na$^+$], 301.06 (100) [M$^+$-B_2H_5], 323.05 (70) [(M-B_2H_6)+Na$^+$]; HRMS (ES$^+$): *m/z*: Calcd. for $C_{18}H_{28}P_2B_2$ [M+Na$^+$]: 351.1750, found 351.1744; calcd. for $C_{18}H_{22}P_2Na$ [(M-B_2H_6)+Na$^+$]: 323.1094, found 323.1089.

2.2 Synthesis of 3

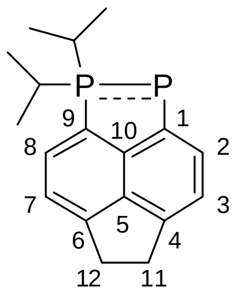

NMR numbering scheme for **3**

To a suspension of **2** (1.8 g, 5.49 mmol) in toluene (40 mL) cooled to -10 °C, Me_2NH (~1.0 g, 22.0 mmol) was added via cannula. Upon addition the suspension turned to a pale red solution and the color intensified to a deep blood red a few seconds later. The reaction was stirred overnight before the solvent was removed in vacuo to give a bright red solid with white crystals of $Me_2NH{\cdot}BH_3$. Toluene (10 mL) was added and the resulting suspension was filtered and the solvent was removed from the filtrate in vacuo. The solid residue was then heated at 70 °C for several hours in vacuo in a flask equipped with an air condenser to sublime off the residual $Me_2NH{\cdot}BH_3$. Compound **3** was isolated as a bright red solid (1.4 g, 4.67 mmol, 85 %). M.p.: 152–154 °C; ^{1}H NMR (400.1 MHz, $CDCl_3$): δ=1.16 (dd, ^3J(H,P)=16.9 Hz, ^3J(H,P)=6.9 Hz, 6H; 2×CH_3), 1.24 (dd, ^3J(H,P)=17.8 Hz, ^3J (H,H)=6.9 Hz, 6H; 2×CH_3), 2.58–2.70 (m, 2H; 2×CH at *i*Pr), 3.26–3.41 (m, 4H; 2×CH_2), 7.10 (dd, ^3J(H,H)=7.1 Hz, ^4J(H,P)=1.4 Hz, 1H; H3), 7.31–7.35 (m, 1H; H7), 7.49 (t, ^3J(H,H)=^3J(H,P)=6.3 Hz, H8), 7.70 (t, ^3J(H,H)=^3J(H,P)=7.5 Hz, H2); ^{13}C{^{1}H} NMR (100.6 MHz, $CDCl_3$): δ=16.8 (br s, CH_3), 17.0 (d, ^2J(C,P)=3.4 Hz, CH_3), 26.1 (dd, ^1J(C,P)=37.6 Hz, ^2J(C,P)=6.8 Hz, C(H) at *i*Pr), 29.9 (s, C11), 31.4 (s, C12), 119.5 (d, ^3J(C,P)=9.4 Hz, C7), 121.6 (d, ^3J(C,P)=8.3 Hz, C3), 122.5 (dd, ^1J(C,P)=52.3 Hz, ^2J(C,P)=13.2 Hz, C9), 125.6 (dd, ^2J(C,P)=32.2 Hz, ^3J(C,P)=13.2 Hz, C8), 129.6 (br s, C2), 136.6 (s, C6), 137.7 (d, ^3J(C,P)=13.5 Hz, C5), 138.3 (dd, ^2J(C,P)=27.0 Hz, ^2J(C,P)=5.8 Hz, C10), 149.0 (dd, ^1J(C,P)=60.1 Hz, ^2J(C,P)=3.8 Hz, C1), 151.2 (d, ^4J(C,P)=2.2 Hz, C4); ^{31}P{^{1}H} NMR (162.0 MHz, $CDCl_3$,):δ=-157.7 (d, P), 76.7 (d, P(*i*Pr)$_2$), ^1J(P,P)=479.6 Hz; Raman (sealed capillary, cm^{-1}): $\tilde{\nu}$=3048 (w), 2938 (m), 2919 (m, νC-H), 1610 (m), 1443 (m), 1410 (vs), 1387 (m), 722 (m); MS (EI$^+$): *m/z* (%): 300.1 (45) [M$^+$], 257.0 (40) [M$^+$-iPr)], 215.0 (100) [(M$^+$-(iPr)$_2$+H)], 152.0 (35) [$C_{12}H_8^+$]; HRMS (EI$^+$): *m/z*: Calcd. for $C_{18}H_{22}P_2$ [M$^+$] 300.1191, found 300.1193.

2.3 Synthesis of 4

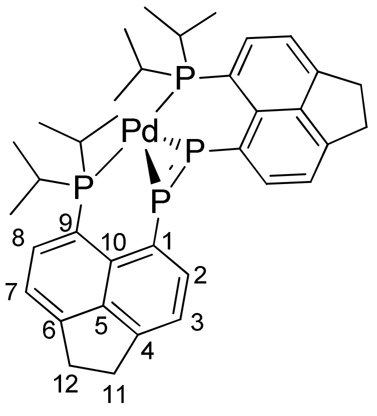

NMR numbering scheme for **4**

To a stirred solution of Pd(PPh_3)$_4$ (385 mg, 0.33 mmol) in toluene (10 mL) cooled to -10 °C, a solution of **3** (200 mg, 0.66 mmol) in toluene (10 mL) was added slowly. The mixture was allowed to warm to RT overnight with stirring. The brown reaction mixture was concentrated to ¼ of its initial volume in vacuo and pentane (10 mL) was added forming a red brown precipitate. The precipitate was collected by filtration, washed with pentane (2× 5mL) and dried in vacuo. Compound **4** was obtained as a dark orange powder (160 mg, 0.23 mmol, 70 %). M.p. decomp. above 190 °C; Anal. calcd. for $C_{38}H_{48}O_{0.5}P_4Pd$ (solvate with ½ a molecule of thf, 743.10 g mol^{-1}): C, 61.42; H, 6.51; found: C, 61.30, H, 6.41; ^{1}H NMR (400.1 MHz, CD_2Cl_2): δ=1.03 (dd, ^3J(H,P)=17.9 Hz, ^3J(H,H)=7.4 Hz, 6H; 2×CH_3), 1.09 (dd, ^3J(H,P)=16.4 Hz, ^3J(H,H)=7.0 Hz, 6H; 2×CH_3), 1.13 (dd, ^3J(H,P)=13.1 Hz, ^3J(H,H)=6.6 Hz, 6H; 2×CH_3) 1.29 (dd, ^3J(H,P)=16.5 Hz, ^3J(H,H)=6.8 Hz, 6H; 2×CH_3), 2.40–2.54 (m, 2H; 2×CH at iPr), 2.79–2.91 (m, 2H; 2×CH at iPr), 3.19–3.35 (m, 8H; 4×CH_2), 7.19 (br d, ^3J(H,H) = 6.8 Hz, 2H; H7), 7.26 (br d, ^3J(H,H)=7.3 Hz, 2H; H3), 7.74 (t, ^3J(H,P)=^3J(H,H)=7.6 Hz, 2H; H8), 8.03–8.08 (m, 2H; H2); ^{13}C{^{1}H} NMR (75.5 MHz, CD_2Cl_2) δ=17.7 (s, 2×CH_3), 19.4–19.6 (m, 2×CH_3), 19.7 (s, 2×CH_3), 22.7 (d, ^2J(C,P) = 9.7 Hz, 2×CH_3), 24.4 (m, 2×C(H) at iPr), 29.9 (s, C11), 30.1 (s, C12), 31.5–32.9 (m, 2×C(H) at iPr), 118.3 (d, ^3J(C,P)=5.9 Hz, C3), 119.7 (s, C7), 126.3–126.6 (m, C1), 131.6 (s, C8), 133.7–140.0 (m, C2), 135.1–137.0 (m, C9), 138.0–138.9 (m, C10), 140.1 (d,

^{3}J(C,P)=7.9 Hz, C5), 145.6 (s, C4), 149.4 (s, C6); $^{31}P\{^{1}H\}$ NMR (121.5 MHz, CD_2Cl_2): AA'XX' spin system, δ 61.2 (m, P), 54.8 (m, P(*i*Pr)$_2$), $^{2}J(P_A,P_X)$=32 Hz, $^{1}J(P_AP_{A'})$=280 Hz, $^{2}J(P_AP_{X'})$=10 Hz, $^{2}J(P_{A'}P_{X'})$=0 Hz; IR (KBr): $\tilde{\nu}$=2921 (s), 2910 (m, νC-H), 1600 (m), 1459 (m), 1380 (m), 840 (s); Raman (sealed capillary): $\tilde{\nu}$=3049 (m), 2922 (m), 2876 (w, νC-H), 1602 (m), 1563 (s), 1443 (m), 1409 (s), 1324 (vs), 824 (m), 718 (vs), 525 (s). MS (EI$^+$): *m/z* (%): 706.2 (60) [M$^+$], 663.1 (100) [M$^+$-(iPr)], 621.1 (60) [M$^+$-(iPr)$_2$+H], 577.0 (98) [(M-(*i*Pr)$_3$]; HRMS (EI$^+$): *m/z*: Calcd. for $C_{36}H_{44}P_4{}^{102}Pd$ [M$^+$] 702.1443, found 702.1444.

3 Crystal Structure Analysis Details

Table 2: Selected bond lengths (Å), angles (°) and dihedral angles (°) for **2**, **3** and **4**·1/2thf. For atom numbering see Figures 1, 2 and 3 in the main text.

Compound **2**

P(1)-P(2)	2.2208(11)		
P(2)-B(1)	1.943(5)	P(2)-B(2)	1.943(4)
P(1)-C(1)	1.791(3)	P(2)-C(9)	1.816(3)
P(1)-P(2)-C(9)	91.05(10)	P(2)-P(1)-C(1)	97.28(10)
B(1)-P(2)-B(2)	118.47(18)	C(1)-P(1)-P(2)-C(9)	11.1

Compound **3** (in square brackets are the respective values for the second molecule in the unit cell)

P(1)-P(2)	2.148(5) [2.147(6)]		
P(1)-C(1)	1.800(13) [1.815(12)]	P(2)-C(9)	1.836(14) [1.828(14)]
P(1)-P(2)-C(9)	90.4(5) [90.2(5)]	P(2)-P(1)-C(1)	100.8(5) [100.8(5)]
C(1)-P(1)-P(2)-C(9)	4.8 [5.7]		

Compound **4**

P(1)···P(2)	3.24	P(3)···P(4)	3.19
P(2)-P(3)	2.123(4)		
P(1)-Pd(1)	2.320(3)	P(4)-Pd(1)	2.322(3)
P(2)-Pd(1)	2.342(3)	P(3)-Pd(1)	2.337(3)
P(1)-Pd(1)-P(4)	134.58(11)	P(2)-Pd(1)-P(3)	53.97(10)
C(1)-P(2)-P(3)-C(21)	164.1		
P(1)-C(9)-C(1)-P(2)	27.2	P(3)-C(21)-C(29)-P(4)	7.8

Table 3: Crystal and structure refinement data for **2**, **3** and **4**·1/2thf.

	**2**	**3**	**4**·1/2thf
Formula	$C_{18}H_{28}B_2P_2$	$C_{18}H_{22}P_2$	$C_{38}H_{48}O_{0.50}P_4Pd$
M_r	327.99	300.32	743.09
Crystal dimensions	0.15 × 0.10 × 0.03	0.03 × 0.03 × 0.03	0.03 × 0.03 × 0.03
Crystal system	Orthorhombic	Monoclinic	Monoclinic
Space group	Pbca	P2(1)/n	P2(1)/c
a [Å]	17.585(4)	16.445(4)	19.874(7)
b [Å]	16.883(4)	11.964(3)	12.668(5)

c [Å]	12.730(3)	18.106(4)	14.050(6)
β [°]	90	113.299(8)	97.408(10)
V [$Å^3$]	3779.4(15)	3271.9(13)	3508(3)
Z	8	8	4
ρ_{calcd} [g cm^{-3}]	1.153	1.219	1.407
μ [mm^{-1}]	0.224	0.254	0.740
$2\theta_{max}$	50.68	50.0	50.76
Measured refln.	22801	19399	22312
Independent refln. (R_{int})	3462 (0.0713)	5689 (0.0942)	6357 (0.1905)
R (F^2, observed data, I>2σ(I))	0.0579	0.1633	0.1095
wR (F^2, all data)	0.1559	0.4231	0.3235
Largest peak/hole [e $Å^{-3}$]	0.73/-0.35	1.50/-0.69	3.22/-1.44

Table 3 lists details of data collections and refinements. All data were collected at 93(2) K using $Mo_{K\alpha}$ radiation (λ = 0.71073 Å) from a high brilliance Rigaku MM007 generator. Data were collected using a Rigaku Mercury ccd detector with ω and φ scans. Intensities were corrected for Lorentz-polarisation and for absorption. The structures were solved by direct methods. Refinements were done by full-matrix least squares based on F^2 using SHELXTL.[2] CCDC 887823 (**2**), 887824 (**3**) and 887825 (**4**) contain the supplementary crystallographic data for this paper. These data can be obtained free of charge via www.ccdc.cam.ac.uk/data_request/cif, or by emailing data_request@ccdc.cam.ac.uk, or by contacting The Cambridge Crystallographic Data Centre, 12, Union Road, Cambridge CB2 1EZ, UK; fax: +44 1223 336033.

4 Computational Part

Additional computational results for 3:

Larger contribution of the ylide resonance form **3** over the doubly bonded form **3’** (see Scheme 2 in main text) is supported by the further computational methods. Thus natural bond orbital (NBO) analysis[3] finds only a single localized P-P σ bond and two lone pairs on the phosphanylidene P atom. The presence of the two lone pairs in **3** is also indicated in a topological ("Atoms In Molecules") analysis[4] of the total electron density, two valence-shell charge concentrations are found above and below the five-membered C_3P_2 ring (as extrema in the Laplacian, see Figure S1). These coincide with high value areas of the electron localisation function (ELF) in the parent H_3PPH.[5] In the canonical Kohn-Sham MOs (Figure S2) the HOMO is essentially a p-orbital on the phosphanylidene P atom with only a slight tendency for delocalisation (both into the acenaphthene π-system and onto the adjacent P atom); the other lone pair is a lower-lying sp^2-type orbital.

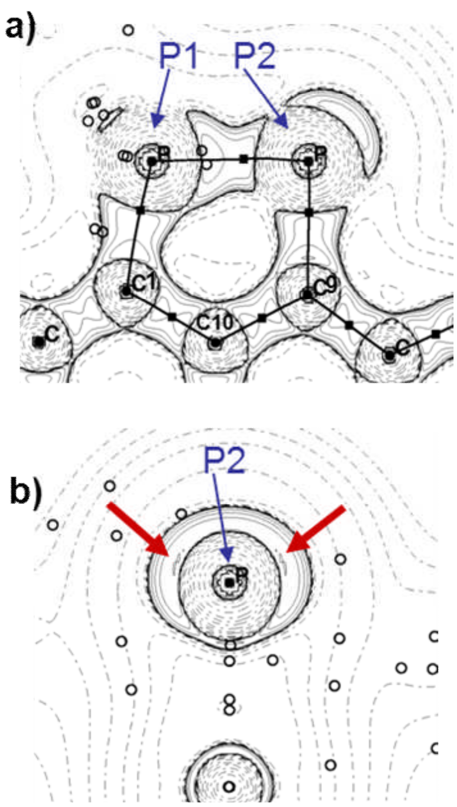

Figure S1 Negative Laplacian of the electron density, $-\nabla^2\rho$, in selected planes of **3** (B3LYP level). a) plane containing the five-membered P_2C_3 ring; b) plane perpendicular to that, cutting through P(2) (phosphanylidene) and the C(1)-C(10) bond. Unfilled circles are projections of nuclei into the plot plane. Bold lines are bond paths in the plot plane with critical points marked as filled squares. Solid lines are in regions where electronic charge is concentrated, dashed lines are in regions where charge is depleted. The two valence-shell charge concentrations on P(2) (phosphanylidene) marking the lone pairs are indicated by red arrows. Key quantities of the critical point between the two P atoms: $\rho = 0.118$ a.u., $-\nabla^2\rho = 0.153$ a.u., ellipticity $\varepsilon = 0.413$. These values are similar to those reported at a similar level for the parent H_3PPH.[5]

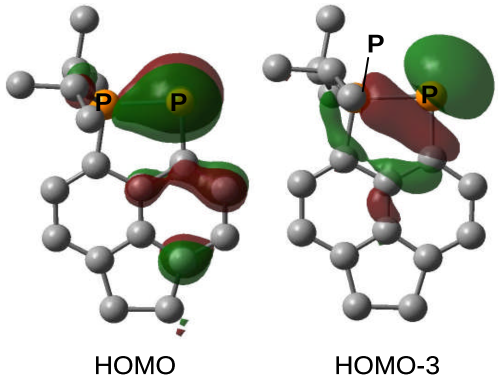

Figure S2 Kohn-Sham MOs in **3** showing lone pair orbitals on phosphanylidene P atom (B3LYP level, H atoms omitted for clarity).

General Computational Details:

Geometries were fully optimized at the B3LYP/6-31G+* level[6] of density functional theory (DFT), together with a fine integration grid (75 radial shells with 302 angular points per shell). Pd was described with the relativistic Stuttgart-Dresden pseudopotential and its associated [6s5p3d] valence basis set.[7] Where available, solid state structures were used as starting points for the optimisations. The nature of the minima was verified by computations of the harmonic frequencies at the same level of theory. Energies are reported at the B3LYP-D3/6-31+G* + ZPE level, i.e. including the empirical dispersion correction by Grimme[8] and zero-point energies, and are corrected for basis-set superposition error (BSSE) according to the Counterpoise method.[9] All computations were performed using the Gaussian09 suite of programs,[10] except for the topological analysis of the B3LYP/6-31+G* electron density of **3**, which used the Morphy program.[11]

References

[1] P. Wawrzyniak, A. L. Fuller, A. M. Z. Slawin, P. Kilian, *Inorg. Chem.* **2009**, *48*, 2500-2506.
[2] G. M. Sheldrick, *Acta Crystallogr. Sect. A* **2008**, *64*, 112 –122.
[3] A. E. Reed, L. A. Curtiss, F. Weinhold, *Chem. Rev*. **1988**, *88*, 899-926.
[4] a) R. W. F. Bader, *Atoms in Molecules*, Oxford Press, New York, **1990**; b) C. F. Matta, R. J. Boyd, *The Quantum Theory of Atoms in Molecules*, Wiley-VCH, Weinheim, **2007**.
[5] A. Sanchez-Gonzalez, S. Melchor, J. A. Dobado, B. Silvi, J. Andres, *J. Phys. Chem. A* **2011**, *115*, 8316–8326.
[6] a) A. D. Becke, *J. Chem. Phys.* **1993**, *98*, 5648-5642; b) C. Lee, W. Yang, R. G. Parr, *Phys. Rev. B* **1988**, *37*, 785-789.
[7] D. Andrae, U. Haeussermann, M. Dolg, H. Stoll, H. Preuss, *Theor. Chim. Acta* **1990**, *77*, 123.
[8] S. Grimme, J. Antony, S. Ehrlich, H. Krieg, *J. Chem. Phys* **2010**, *132*, 154104.
[9] S. F. Boys, F. Bernardi, *Mol. Phys.* **1970**, *19*, 553-566.
[10] M. J. Frisch, G. W. Trucks, H. B. Schlegel, G. E. Scuseria, M. A. Robb, J. R. Cheeseman, G. Scalmani, V. Barone, B. Mennucci, G. A. Petersson, H. Nakatsuji, M. Caricato, X. Li, H. P. Hratchian, A. F. Izmaylov, J. Bloino, G. Zheng, J. L. Sonnenberg, M. Hada, M. Ehara, K. Toyota, R. Fukuda, J. Hasegawa, M. Ishida, T. Nakajima, Y. Honda, O. Kitao, H. Nakai, T. Vreven, J. Montgomery, J. A., J. E. Peralta, F. Ogliaro, M. Bearpark, J. J. Heyd, E. Brothers, K. N. Kudin, V. N. Staroverov, R. Kobayashi, J. Normand, K. Raghavachari, A. Rendell, J. C. Burant, S. S. Iyengar, J. Tomasi, M. Cossi, N. Rega, N. J. Millam, M. Klene, J. E. Knox, J. B. Cross, V. Bakken, C. Adamo, J. Jaramillo, R. Gomperts, R. E. Stratmann, O. Yazyev, A. J. Austin, R. Cammi, C. Pomelli, J. W. Ochterski, R. L. Martin, K. Morokuma, V. G. Zakrzewski, G. A. Voth, P. Salvador, J. J. Dannenberg, S. Dapprich, A. D. Daniels, Ö. Farkas, J. B. Foresman, J. V. Ortiz, J. Cioslowski, D. J. Fox, Gaussian 09, Rev. A.02, Wallingford CT, **2009**.
[11] P. L. A. Popelier, *Comput. Phys. Commun* **1996**, *93*, 212-240.
